# Patients’ perceptions of physical activity and cardiac rehabilitation after spontaneous coronary artery dissection (SCAD): a focus group study in Sweden

**DOI:** 10.1136/bmjopen-2026-116811

**Published:** 2026-06-17

**Authors:** Karin Alpkvist, Sofia Stenberg, Sofia Sederholm Lawesson, Sabina Borg, Eva Swahn, Ingela Thylén

**Affiliations:** 1 Department of Health, Medicine and Caring Sciences, Linköping University, Linköping, Sweden; 2 Clinical Department of Cardiology, Region Östergötland, Linköping, Sweden

**Keywords:** QUALITATIVE RESEARCH, Self-Management, Coronary heart disease, Ischaemic heart disease, Myocardial infarction

## Abstract

**Objectives:**

Spontaneous coronary artery dissection (SCAD) is a rare cause of acute coronary syndrome mainly affecting younger to middle-aged women. Physical activity has been discussed as a potential trigger for SCAD. This study aimed to explore the perceptions of physical activity among patients and their experiences of cardiac rehabilitation following the event.

**Design:**

Qualitative descriptive study using digital focus groups and inductive qualitative content analysis.

**Setting:**

Six digitally conducted focus groups (Zoom) in Sweden.

**Participants:**

Twenty-six women and three men (median age 57 years) who had experienced SCAD within the past 3 months to 3 years were recruited via a national SCAD peer-support group and eight university hospitals.

**Results:**

Participants continually re-negotiated their bodily capacity after SCAD, marked by fear, uncertainty and a loss of bodily trust. Difficulty interpreting bodily signals and fear of physical strain often led to avoidance of physical activity. Recovery was experienced as slow and unpredictable, reinforced by inconsistent or insufficient advice from healthcare professionals. Limited follow-up and lack of SCAD-specific rehabilitation contributed to feelings of isolation and abandonment. Despite these challenges, participants gradually developed personal strategies to rebuild strength and confidence. Peer interaction and shared experiences contributed to emotional safety, motivation and hope during rehabilitation.

**Conclusion:**

Post-SCAD rehabilitation should address both physical and emotional recovery through coherent, person-centred follow-up with individualised guidance and opportunities for peer support to reduce uncertainty and support long-term recovery.

**Trial registration number:**

Not applicable.

**STRENGTHS AND LIMITATIONS OF THIS STUDY**

- Purposive sampling with maximum variation across sex, age and geography captured diverse experiences of post-spontaneous coronary artery dissection (SCAD) physical activity and rehabilitation.
- Digital focus groups enabled national recruitment and facilitated participation from multiple regions.
- Use of inductive qualitative content analysis and team discussions supported analytic rigour and transparency.
- Recruitment partly via a peer-support group may have attracted participants with higher engagement or stronger views on physical activity.
- The wide time range since the SCAD event (3 months to 3 years) may have influenced recall, although it also enabled insights across recovery phases.

## Introduction

 Spontaneous coronary artery dissection (SCAD) is a rare cause of acute coronary syndrome (ACS), resulting from epicardial coronary artery dissection and lumen obstruction due to an intramural haematoma or intimal tear.^1 2^ The condition predominantly affects young to middle-aged women, who represent about 80%–90% of cases, with a mean age at onset of ~50 years.^3 4^ Estimates suggest that SCAD accounts for 1%–4% of all ACS presentations,^25^,^7^ whereas it accounts for up to 35% of presentations among women younger than 50 years.^3 5^ Due to its rare occurrence, SCAD is frequently underdiagnosed and often initially misclassified.^2^

Unlike other ACS patients, those with SCAD often lack traditional cardiovascular risk factors such as smoking, hyperlipidaemia and diabetes,^8^ and it is instead linked to the vasculopathy fibromuscular dysplasia. SCAD is considered to have multifactorial causes, with emotional or physical stressors commonly reported as triggers.^1^ While intense or isometric physical exertion is frequently linked to SCAD in men, and psychological stress tends to be more prevalent among women,^9^ the relationship between physical activity and SCAD remains unclear. Although a potential link has been suggested,^10 11^ current evidence does not support a definitive causal connection.

Following ACS, many patients develop a fear of engaging in physical activity and exercise, usually referred to as kinesiophobia.^12^ Cardiac rehabilitation, a multidisciplinary intervention including physical training, is proven to reduce mortality, reduce cardiovascular risk factors, improve exercise capacity and enhance quality of life.^13^,^15^ International guidelines strongly recommend cardiac rehabilitation after ACS due to plaque rupture.^14^ However, it remains unclear how SCAD patients engage in cardiac rehabilitation, what guidance they receive and what a SCAD-specific cardiac rehabilitation programme should include. A recent systematic review of SCAD survivors concluded that traditional cardiac rehabilitation programmes fail to meet their needs and that SCAD patients are advised to exercise at lower intensities than other ACS patients.^4^ Although this review highlighted the limitations of traditional cardiac rehabilitation programmes for SCAD survivors, there remains a lack of in-depth understanding of how these individuals perceive physical activity and the support they receive from healthcare professionals. By conducting a qualitative study, we aimed to address this gap by exploring the experiences and perspectives of patients with SCAD on physical activity and cardiac rehabilitation resources available for them, thereby informing future programme development.

## Methods

### Study design

This qualitative descriptive study used focus groups to facilitate interaction and shared experiences, enabling the identification of diverse perspectives and previously unnoticed aspects, and was underpinned by inductive qualitative content analysis.^16^

The study adhered to the Consolidated criteria for Reporting Qualitative research (COREQ) checklist for qualitative research reporting.^17^

## Sampling method and participants

Eligible participants were Swedish-speaking adults (≥18 years) who had experienced SCAD within the past 3 months to 3 years. Recruitment used purposive sampling via a SCAD support group (Facebook) and eight university hospitals in Sweden. For hospital-based recruitment, clinicians informed patients with a known SCAD diagnosis about the study during routine outpatient visits and provided study information and contact details. Interested individuals contacted the first author by email. Oral study information was provided, including details about audio and video recording, anonymisation and confidentiality. Consent forms were mailed to those who agreed to participate. SCAD diagnoses were verified through medical record review with the written consent of the participants. When access to electronic records was not possible, participants provided copies from their medical journal of relevant medical documentation. For hospital-recruited participants, diagnosis was presumed accurate. Focus groups were scheduled at times agreed on by all participants after several alternative dates had been proposed. Purposive sampling with maximum variation in sex, age and geographic location ensured diverse perspectives. Sample size and composition followed recommendations by Krueger and Casey.^16^ Participants were recruited consecutively as groups were scheduled. In total, 30 individuals with verified SCAD were approached and agreed to participate; one later withdrew due to illness. Data saturation was reached after the sixth focus group. Sociodemographic characteristics are presented in table 1.

**Table 1:** Sociodemographic characteristics

	Group 1 (n=5)	Group 2 (n=5)	Group 3 (n=5)	Group 4 (n=4)	Group 5 (n=5)	Group 6 (n=5)
Age (years), median (range)	63 (55–71)	57 (32–61)	53 (38–59)	51 (34–77)	58 (48–63)	57 (55–64)
Gender, female	5	4	5	3	5	4
Time since SCAD ((months), median (range))	20 (7–30)	11 (5–33)	16 (7–32)	9.5 (4–36)	17 (3–24)	8 (7–36)
*Region of residence* Southern Sweden Central Sweden Northern Sweden	0 4 1	1 3 1	1 4 0	0 3 1	0 3 2	1 4 0

SCAD, spontaneous coronary artery dissection.

### Data collection

#### Focus groups

Before conducting the focus groups, a pilot session with patients with experience of SCAD was carried out to test the interview guide for clarity, relevance and sensitivity. As no major revisions were needed, the pilot was included in the final dataset.

Digital interviews via Zoom were conducted between June 2024 and January 2025 to ensure geographic diversity and account for regional variations in SCAD follow-up care.

Five groups involved five participants each, and one group had four participants. Interviews were conducted by two female PhD students and medical doctors, one acting as a moderator (KA) and the other as an observer (SS). Neither had clinical involvement with the participants or prior experience of conducting qualitative focus groups; both prepared for the role through study-specific training and familiarisation with qualitative interview methodology.

Before each interview, participants received oral information about the study and guidelines, including voluntariness, the right to decline sensitive topics, respectful interaction and confidentiality. Interviews began with the moderator introducing the topic. The moderator and observer also presented their professional backgrounds as medical doctors and PhD students and informed the participants about the purpose of the study. Participants then introduced themselves. The observer took brief notes and summarised each interview. A semi-structured interview guide was used throughout the process (table 2).

**Table 2:** Interview guide

Question category	Interview guide questions
Introductory questions	“What does physical activity mean to you?”
Transition question	“What factors affect your physical activity today in comparison to prior to your SCAD diagnosis?”
Key questions	“What challenges do you experience in being physically active after your SCAD diagnosis?” “What could have made it easier for you to engage in physical activity post-SCAD?” “How do you perceive the advice provided by healthcare professionals regarding physical activity after SCAD?” “If you could speak freely, how would you like the healthcare system to support physical activity following a SCAD diagnosis?”
Closing question	“Is there anything else regarding physical activity after SCAD that you would like to share or that we have not addressed?”

SCAD, spontaneous coronary artery dissection.

Each interview lasted between 40 min and 90 min, was conducted in Swedish via Zoom, audio-recorded and video-recorded, and transcribed verbatim by the moderator for subsequent analysis.

### Data analysis and methodological rigour

Data was analysed using inductive qualitative content analysis, which is suitable for descriptive analyses of interview data and underexplored phenomena.^18 19^ Data was analysed manually. Each focus group was treated as the unit of analysis, and a manifest approach was applied. In the preparation phase, transcripts were repeatedly read for overall understanding. In the organising phase, meaning units were identified, condensed and coded, then grouped into subcategories based on semantic similarity and abstracted into broader categories. Data coding was conducted by the first author (KA) which were subsequently reviewed, challenged and refined through systematic comparison with interpretations made by the other researchers. Quotations illustrated each subcategory and enhanced transparency,^20^ with source identifiers linked to focus group number (eg, FG1). Although individual experiences varied to some extent, the categories presented reflect shared patterns across participants, rather than isolated or individual perspectives. Table 3 provides an example of the analytical process.

**Table 3:** Example of the analytical process

Meaning unit	Condensed meaning unit	Code	Subcategory	Category
“I don’t really know how to describe it, but it’s something that’s always there… a kind of worry. Like, what if I do something, what if I prune those branches, and then maybe a SCAD appears again? It’s always with me.”	A constant, underlying worry that certain activities might trigger a new SCAD event	Fear of causing a new SCAD episode	Fear of physical strain and uncertainty of bodily signals	Re-negotiating one’s bodily capacity

SCAD, spontaneous coronary artery dissection.

To ensure methodological rigour, Lincoln and Guba’s trustworthiness framework was applied.^21^ Qualitative content analysis was chosen as it suits focus group data and allows systematic categorisation of the experiences of patients while remaining close to the manifest content, enabling a structured analysis of perspectives on physical activity and rehabilitation after SCAD.^16 19^ The interview guide was developed based on the aim of the study and pilot-tested for clarity and relevance.^18^ Credibility was supported by including participant quotes in the results to illustrate interpretations and category development.^20^ Transferability was addressed through detailed descriptions of context, sampling and participant characteristics. Analytical triangulation strengthened dependability and confirmability.^19 21 22^ Reflexivity was maintained by acknowledging the background and preunderstanding of researchers.

### Patient and public involvement

Although patients were not directly involved in the conduct of this study, its focus was shaped by recurring discussions with SCAD patients in clinical settings, annual patient meetings and interactions with patient representatives within a national SCAD peer-support group who also informed the relevance of the research questions.

## Results

In total, 12 subcategories and five overarching categories emerged (table 4). Together, these categories illuminate how physical activity after SCAD is not merely a behavioural adjustment, but a deeply embodied and socially embedded process of recovery, negotiation and meaning making.

**Table 4:** Categories and subcategories

Category	Subcategory
Re-negotiating bodily capacity	Fear of physical strain and uncertainty of bodily signals
	A slow and unpredictable physical recovery
Navigating an unclear informational landscape	Conflicting advice on physical activity
	Lack of concrete and practical guidance
	Personal efforts to seek out information
Seeking support within a complex healthcare context	Lack of continuity and belonging in rehabilitation
	Care is not tailored to individual needs
	Valuing access to professional support
Creating meaning through connection and recognition	Validation through meeting others with similar experiences
	Sense of safety in not being alone in one’s situation
Regaining control over everyday physical activity	Adjusting exercise to daily condition and capacity
	Physical activity as a pathway to recovery and normality

### Re-negotiating bodily capacity

This category explores the internal struggle of participants to interpret and trust bodily signals, often marked by fear, uncertainty and a sense of vulnerability.

#### Fear of physical strain and uncertainty of bodily signals

Groups discussed a persistent fear of overstraining the heart following SCAD, which often led to avoidance of physical activity. This fear was closely linked to a heightened awareness of bodily sensations and uncertainty about how to interpret them. Groups discussed hesitation to engage in exercise or even routine activities, concerned physical exertion might provoke another cardiac event. One participant described how this fear caused her to stop exercising before reaching any level of intensity:

But I always stop… I mean, before I even get to that point… because I just don’t dare, I don’t dare… to risk it happening again, you know… (FG4)

In addition to this fear, groups described increased sensitivity to bodily signals, particularly changes in heart rhythm or intensity. This hypervigilance led to anxiety and uncertainty about what was normal or dangerous. One participant reflected on how her perception had changed since the SCAD event:

I’m much more aware now in a way I wasn’t before, like… back then, I didn’t really notice how my heart was beating or how I was feeling in that sense. So, I think that affects me a lot… and at the slightest sensation, I feel scared, like, when I notice that something feels off, which I probably wouldn’t have noticed before. But now, I notice everything, kind of… (FG4)

#### Slow and unpredictable physical recovery

Some groups described the physical recovery process after SCAD as slower and more unpredictable than expected. There was a shared sense that their bodies no longer responded to physical activity in the same way as before the event. Despite engaging in rehabilitation or attempting to resume previous levels of activity, many experienced lingering fatigue, reduced stamina and frustration over the inconsistency of their physical condition. One participant reflected on how the recovery process had been both physically and emotionally challenging:

I mean, it took a long time to get my strength back… but I was also really scared… I was… and of course that led to quite a bit of weight gain… I’m trying to work on that now, but I still really relate to that feeling – that the energy just isn’t there in the same way anymore… And it’s been two years since I had my SCAD. (FG5)

The passage of time did not automatically lead to the anticipated return to normality, highlighting the complex and often discouraging nature of the recovery process.

### Navigating an unclear informational landscape

This category explores efforts of participants to make sense of inconsistent or insufficient physical activity guidance, focusing on the clarity, content and consistency of information from healthcare professionals.

#### Conflicting advice on physical activity

Groups discussed receiving inconsistent or even contradictory guidance from different healthcare professionals regarding physical activity advice after SCAD. While some were advised to be cautious and follow strict limitations, others were encouraged to return to activity without restrictions. This inconsistency created confusion and uncertainty, particularly in the early stages of recovery. In addition to personal experiences, groups also shared how fellow SCAD patients had received entirely different recommendations, further amplifying the sense of unpredictability in post-SCAD care. One participant reflected on the frustration of being presented with very specific written advice, only to later be told to disregard them:

I feel like we’ve been given mixed messages. First, you get a list that says things like… If you’re going to exercise, it should be with lighter weights and lots of repetitions. You shouldn’t lift more than 9 kg, or whatever it said… There were four or five points like that, all on a piece of paper. And then when you show up, they tell you… Oh, just go for it! (FG5)

The lack of consistency in formation and advice was a recurring discussion across groups. This variation became especially evident in discussions involving participants from different regions of Sweden, where differences in access to rehabilitation resources and professional recommendations were brought to light. One participant reflected on how the rehabilitation process was complicated not only by fear, but also by the conflicting messages received:

I had never really thought that I could have a heart attack… That possibility just didn’t exist in my mind, at least – not that something like that would happen to me. And then another challenge with physical activity is this fear, combined with getting completely different messages from all directions… There should be something created that people can actually relate to—something that applies across Sweden, at all hospitals. (FG5)

#### Lack of concrete and practical guidance

Groups expressed frustration about the lack of clear and practical guidance from healthcare professionals regarding physical activity after SCAD. Instead of receiving clear and structured recommendations, a common piece of advice reported was to ‘listen to your body’. While this suggestion was likely intended to encourage self-awareness and caution, many participants found it vague and difficult to act on, particularly in the context of recovering from a sudden and life-threatening cardiac event.

The lack of specific thresholds or physiological markers, such as heart rate or blood pressure limits, left some participants feeling uncertain and unconfident in their ability to safely resume physical activity. As one participant explained:

And I feel safer having concrete advice, like about heart rate or blood pressure… At the hospital they say, ‘you should listen to your body,’ but I find it really hard to know where the limit is. So, it’s easier for me to have numbers—like heart rate—to go by. (FG3)

The absence of nationally consistent guidelines or care pathways further contributed to this sense of uncertainty. While individualised care was acknowledged as important, participants expressed a need for a baseline of standardised information and practices that could offer a more coherent and equitable foundation for post-SCAD rehabilitation nationwide, as discussed in this focus group:

Develop national guidelines for SCAD, in order to get equal care, regardless of geographic location. (FG4)

#### Personal efforts to seek out information

In the absence of clear or tailored guidance following their SCAD event, some groups discussed actively seeking information on their own. This involved Internet searches, reading medical websites and engaging with online forums or support groups where they could connect with others who had experienced SCAD. This was unfortunately not always experienced as helpful or reassuring. While some participants appreciated the opportunity to share experiences and learn from others, many found the information to be overwhelming, inconsistent or anxiety-inducing. The unfiltered nature of online content, combined with the emotional vulnerability following a cardiac event, sometimes led to increased fear rather than clarity. One participant described how these efforts led to feelings of fear and discouragement rather than motivation:

Then I read quite a lot and talked to people… There was a bit of fear when reading… I wasn’t really motivated to exercise… It was more discouraging, that it was taking so long… There was some fear when reading about these things. (FG6)

Several groups expressed a desire for curated, trustworthy resources that could offer both medical accuracy and emotional reassurance. The need to seek peer-led information highlights the lack of accessible, evidence-based information tailored to SCAD patients. Without such resources, individuals were left to navigate a complex and often contradictory landscape of advice, which could hinder rather than support their recovery process.

### Seeking support within a complex healthcare context

This category explores the experiences of participants regarding healthcare organisation and delivery, including continuity and relational aspects of care, access to support and perceived individualisation.

#### Lack of continuity and belonging in rehabilitation

Groups discussed how the post-SCAD period was characterised by fragmented care and a sense of being left on their own in the rehabilitation process. The degree of follow-up varied substantially depending on geographical location and healthcare facility. While some received structured and multidisciplinary follow-up, including consultations with physicians and physiotherapists, others reported minimal or virtually no specialised support. This inconsistency in continuity of care was perceived as a significant gap in the recovery process and contributed to feelings of abandonment and uncertainty. One participant described her experience of inadequate follow-up support:

But my body has refused. And no help at all from the hospital… I had a follow-up call with a nurse at the cardiology clinic, but no other support after that, it was just handed over to the primary care. (FG1)

The transition from specialised cardiac care to primary care was often experienced as abrupt, with limited communication between healthcare providers, thereby reinforcing the sense of uncertainty and lack of confidence among patients regarding the continuity of their care. This was further amplified by the limited and, in some cases, absent knowledge about SCAD among primary care providers. Groups discussed that their needs did not fit within the existing cardiac rehabilitation framework. Standard programmes, such as the heart school, were often perceived as irrelevant or even alienating, as they were designed for patients with more common cardiac conditions. This mismatch reinforced a sense of isolation and being misunderstood.

As one participant expressed:

I was really upset after attending heart school 12 times… I thought to myself, here I am, a living example that no one knows anything about my condition… (FG1)

The absence of tailored follow-up and the lack of peers with similar experiences contributed to a feeling of loneliness in the recovery process.

#### Care is not tailored to individual needs

Beyond the content of advice, groups expressed frustration over what they perceived as a lack of individualised care following their SCAD event. Despite the diversity in the backgrounds, lifestyles and prior activity levels of patients, the healthcare response was frequently experienced as standardised and generic. For those who had previously been highly physically active or engaged in competitive sports, the advice provided was often perceived as overly cautious.

The absence of structured individual assessments or meaningful discussions regarding the previous physical activity and psychological profile of patients meant that care was not adequately adapted to their specific needs and circumstances. This lack of personalisation risked engagement in rehabilitation. Participants emphasised that rehabilitation must reflect individual life contexts and previous experience, in order to feel meaningful and motivating. One participant reflected on this lack of personalisation:

No one asks anything… I’ve never been asked, like, how has it been for you? From that, you could also tell if this person maybe needs to be held back a little… If you’re really competitive, for example… (FG5)

A more person-centred approach to post-SCAD care, incorporating individualised risk assessment, lifestyle context and psychological factors in the planning of follow-up and rehabilitation, was discussed by focus groups.

#### Valuing access to professional support

Participants described a sense of safety and control when participating in supervised cardiac rehabilitation sessions. Having access to healthcare professionals many times contributed to feelings of reassurance, particularly in the early phases of recovery when uncertainty and fear of recurrence were prominent. The presence of trained staff and the fact that they could monitor vital signs such as blood pressure and heart rate as well as having the possibility to ask questions were seen as key factors in creating a secure atmosphere. As one participant expressed:

Yes, I felt good about going to rehab because I had access to staff, and I could check my blood pressure and pulse… so it felt safe to be there. (FG2)

This sense of safety eased the rehabilitation process and helped participants regain confidence in their physical capacity. In contrast to exercising alone, the monitored setting reduced anxiety, increased motivation and eased the fear of triggering another cardiac event.

The opportunity to share progress and challenges in a group setting also contributed to increased confidence and trust in the rehabilitation process. One participant talked about the significance of this type of support, even mentioning that structured group-based activities would have been the first thing to prioritise in the rehabilitation process:

I really would have wanted group activities, like the ones you’re talking about… I wanted that desperately… but there was no offer or even any information about it… so that would definitely have been my first priority. (FG1)

### Creating meaning through connection and recognition

This category explores how social interaction and shared experiences with peers contributed to emotional safety, motivation and hope throughout the rehabilitation process.

#### Validation through meeting others with similar experiences

Groups discussed the importance to connect with others who had experienced SCAD and talked about the importance of peer support in the recovery process. Many described a sense of loneliness and isolation following their SCAD event, partly due to the rarity of the condition and the lack of public and professional awareness. Meeting others with similar experiences was seen as a way to exchange coping strategies and to feel less alone in the recovery process. One of the participants reflected on this need for peer support:

You’d really want to meet others who have SCAD too, because that’s where you might find some kind of… recognition… that maybe some of us experience things in a certain way, and others differently… That even within the SCAD group, we could sort of form subgroups… where you find, like… you and I are going through similar things. (FG4)

Identifying similarities in experiences, feelings and challenges were discussed as important. Peer support was described as potentially more meaningful than support from general cardiac patient groups, where the specific context of SCAD was often missing. In addition to in-person encounters, participants also described connecting with others through digital platforms and online communities. These spaces offered opportunities to share personal experiences, challenges and progress, and were sometimes the only source of peer interaction available.

#### Sense of safety in not being alone in one’s situation

Participants discussed emotional reassurance that came from realising they were not alone in having experienced a SCAD event. Given the rarity of the condition, many described initially feeling isolated and uncertain. Seeing or hearing about others recovering from SCAD provided a sense of safety and validation that could increase both motivation and hope for recovery. Participants also expressed that seeing others share their recovery journeys, whether in person or online, helped normalise their own fears and uncertainties. Observing the progress of others fostered a sense of hope and made it easier to trust in their own capacity to recover. One participant described seeing another SCAD survivor resume running after their SCAD event:

I only started to feel more confident when I saw someone who had suffered a SCAD during a running race and then ran the same race the following year. And that made me think - oh, maybe I can do this too. So, if that kind of support had been available from the start… I don’t really know how it would work, through the healthcare system, but some kind of national support structure, just because it’s such a rare condition, I really think that would be needed. (FG2)

### Regaining control over everyday physical activity

This category explores how participants gradually regained a sense of control and agency through personalised strategies, experimentation and the re-establishment of physical routines. Physical activity was perceived not only as a means to rebuild physical strength but also as a pathway towards emotional recovery and a redefinition of normality.

#### Adjusting exercise to daily condition and capacity

Participants who had previously engaged in regular or high-intensity physical activity described the need to adapt their approach to exercise following their SCAD event. For many, physical activity had been a central part of their identity and well-being prior to the incident. But following SCAD, uncertainty about physical limits, fear of recurrence and lack of clear guidance made their response to physical activity more tentative and cautious. Adjustments included decreasing the intensity of workouts, selecting safer activities and paying more attention to body signals, which was seen as a constructive way to move forward. These accommodations often required acceptance, patience and a redefinition of what it meant to be active. As one participant shared:

I’ve also always been very active… not an elite athlete, but I’ve always really enjoyed moving and exercising in different ways. I’ve kept in shape through both going to the gym and… running a lot and, like… so it’s been quite an adjustment to try and find something that feels safe. (FG5)

While some participants described a sense of loss related to their previous capacity for high-intensity exercise, others described a more adaptive and accepting outlook. Discovering new forms of physical activity was not necessarily viewed as something bad, but rather it offered alternative ways to experience physical activity and maintain well-being. One participant reflected on this shift:

I also used to train much more intensively before… when I was running, and also with heavier weights—strength training and so on… I’ve had to cut back on that now. High-intensity workouts are no longer part of my routine… but I’ve learned that lower-intensity works too. What I’ve noticed is that I might not run as fast anymore, but I run longer than before without getting completely exhausted. So yes… that’s my limitation, but it also has its charm, in a way… (FG3)

This openness to change and experimentation was described as an important part of regaining control and confidence in physical capacity.

#### Physical activity as a pathway to recovery and normality

Some groups emphasised the significance of gradually reintroducing daily physical activities that had previously been part of their routine. These activities, such as running at a slower pace, cycling or swimming, were not only physically beneficial but also emotionally meaningful, symbolising progress and a return to normal life. The initial period after SCAD was often marked by uncertainty and fear. Over time, groups discussed how slowly resuming familiar activities helped rebuild trust in their bodies in the recovery process. One participant shared:

In the beginning, it was a lot about not daring, because I didn’t really know how much I was allowed to do… but now I’ve started cycling to work again, and that’s something I used to do before as well. (FG2)

This gradual reintegration of physical routines was described as empowering, helping participants reconnect with aspects of their identity and everyday life that had been disrupted by the SCAD event.

## Discussion

This study highlights how individuals recovering from SCAD navigate a complex interplay between bodily awareness, healthcare guidance and social support in their efforts to re-engage with physical activity. Participants described a heightened sensitivity to bodily sensations and an ongoing fear of recurrence, often leading to hesitation or avoidance of exercise. These findings echo previous studies showing that anxiety about exertion and uncertainty about bodily cues are common among SCAD survivors.^23^ However, our findings extend this knowledge by showing that fear of physical strain was closely tied to a disrupted sense of bodily trust. Previously familiar sensations such as changes in heart rhythm, breathlessness or fatigue had become ambiguous and difficult to interpret, shaping everyday decisions about movement. This prolonged ambiguity illustrates how recovery involves renegotiating bodily confidence rather than simply regaining physical capacity.

Another central finding was the lack of consistent and concrete guidance from healthcare professionals. Participants reported receiving conflicting advice regarding physical activity, ranging from strict limitations to unrestricted encouragement, which created confusion and undermined trust in the rehabilitation process. Similar concerns have been raised in earlier studies.^24^ A commonly reported example was the advice to ‘listen to your body’, which was experienced as vague and difficult to act on, particularly in the context of a rare and poorly understood condition. Our findings further show that inconsistencies were not only interpersonal but also systemic, with notable variation between regions, hospitals and professional groups. This variability shaped the sense of safety among patients and contributed to uncertainty about which recommendations could be trusted. Calls for nationally coherent guidance by participants highlight how inconsistent information was experienced as a barrier to recovery and a source of perceived inequity in care.

Our findings also underscore the importance of continuity and person-centred care, consistent with previous qualitative research among SCAD survivors.^25^ Participants described abrupt transitions from specialised cardiac care to primary care, often without structured follow-up or tailored support. This fragmentation of care has been identified as a barrier to recovery in other qualitative studies on SCAD.^26^ Moreover, the lack of individualised assessments—particularly regarding previous activity levels and psychological needs—was seen as a missed opportunity to foster engagement and confidence in rehabilitation. However, our findings extend previous knowledge by showing that continuity was experienced not only as coordination of services but as a need for relational anchoring. Participants sought a sense of being ‘held’ within the healthcare system, and when this safety net disappeared during transitions, it contributed to feelings of abandonment and uncertainty. This was reinforced by the perception that SCAD did not fit within existing rehabilitation structures and that primary care lacked the necessary expertise.

Social connection also emerged as a key source of emotional reassurance and motivation in our study. Participants valued meeting others with similar experiences, in person or online. Peer support was seen as more meaningful than general cardiac rehabilitation, where SCAD-specific context was often lacking. These findings align with previous research showing that SCAD-specific peer networks reduce isolation and validate experiences.^27^ Our findings further show that peer support played a central role in helping participants make sense of their recovery. Encounters with other SCAD survivors provided interpretive guidance, normalised fears and offered embodied examples of what recovery could look like, helping to rebuild confidence in their own capacity to recover.

Importantly, participants described how regaining control over physical activity involved a process of experimentation, adaptation and gradual reintroduction of familiar routines. While some mourned the loss of high-intensity exercise, others embraced new forms of movement that felt safer and more sustainable. This adaptive approach reflects themes of resilience and self-efficacy, noted in previous studies exploring recovery after SCAD.^23^ Our study adds that these adaptations often occurred through micro-adjustments embedded in daily life rather than through formal rehabilitation structures. Participants developed personalised strategies, adjusted pace, modified routines and redefined what ‘counts’ as physical activity, which allowed them to regain agency despite uncertainty. This flexible, context-sensitive approach suggests that SCAD survivors may benefit from rehabilitation frameworks that explicitly legitimise and support such adaptive strategies.

### Implications for practice, policy and research

By illuminating the processes through which patients negotiate bodily trust, construct their own informational frameworks, seek relational continuity and create meaning through peer connection, this study contributes novel insights into the lived complexity of SCAD recovery. These findings highlight the need for rehabilitation models that address not only physical safety but also the interpretive, relational and existential dimensions of recovery.

For clinical practice, the results point to the importance of structured and consistent post-SCAD support, including clear, individualised guidance on physical activity and attention to emotional recovery. More coherent care pathways with defined points of contact and SCAD-specific competence may strengthen continuity and reduce the sense of abandonment described by participants. Peer support should also be considered a valuable component of rehabilitation, given its role in meaning-making and emotional reassurance.

From a policy perspective, the findings underscore the need for SCAD-specific recommendations within cardiac rehabilitation frameworks, ensuring that rare-disease expertise is accessible across care levels. Future research should evaluate tailored rehabilitation programmes, including digital solutions and structured peer-based interventions, and examine how these can best support the bodily confidence, informational needs and long-term recovery of patients.

### Limitations and strengths

While the number of participants was relatively small, the sample size was appropriate for a qualitative focus group design,^16^ and data saturation was considered reached after the sixth interview. While some focus groups lacked diversity in age and gender, this mirrors the epidemiological profile of SCAD, which predominantly affects women of younger to middle age.^3 10^ Nevertheless, the limited heterogenicity may have narrowed the spectrum of perspectives.

Furthermore, participants with a particular interest in physical activity may have been over-represented, potentially introducing bias but also offering detailed insights. Lack of socioeconomic and demographic data limits assessment of diversity. Variation in time since SCAD (3 months to 3 years) may have affected recall, while also providing insight into recovery over time.

Finally, focus groups were conducted online due to the rarity of SCAD and the geographic dispersion of participants across Sweden. While this facilitated participation and reduced logistical barriers,^28^ it may have influenced group dynamics, such as turn-taking and spontaneity.^29^ Participants nevertheless appeared comfortable and engaged throughout.

## Conclusion

This study contributes to a growing body of knowledge on SCAD by illuminating how survivors navigate physical recovery in the absence of tailored support. Our findings highlight recovery after SCAD as an ongoing process of re-negotiating bodily trust, where uncertainty about physical strain and bodily signals shapes engagement in physical activity. The study also demonstrates the important role of peer support in providing emotional reassurance, validation and hope during recovery. The findings suggest that rehabilitation should not only address physical capacity but also emotional safety, individual context and social connection. A more coherent and person-centred approach to SCAD care may enhance recovery outcomes and reduce the burden of uncertainty that many patients face.

## Data Availability

Data are available upon reasonable request.
