# Supplementary material for: Patients’ perceptions of physical activity and cardiac rehabilitation after spontaneous coronary artery dissection (SCAD): a focus group study in Sweden
Source: BMJ Open. 2026 Jun 17;16(6):e116811. doi: 10.1136/bmjopen-2026-116811 (PMC13289010; doi:10.1136/bmjopen-2026-116811)
Supplement: Supplementary file 1 [file bmjopen-2026-116811.reviewer_comments.pdf]

PEER REVIEW HISTORY

BMJ Open publishes all reviews undertaken for accepted manuscripts. Reviewers are asked to complete a checklist review form and are provided with free text boxes to elaborate on their assessment. These free text comments are reproduced below.

ARTICLE DETAILS

Title (Provisional)

Patients' perceptions of physical activity and cardiac rehabilitation after spontaneous coronary artery dissection (SCAD): a focus group study in Sweden

Authors

Alpkvist, Karin; Stenberg, Sofia; Sederholm Lawesson, Sofia; Borg, Sabina; Swahn, Eva; Thylén, Ingela

---

VERSION 1 - REVIEW

**Reviewer**	**1**
**Name**	**Hanson, Coral**
**Affiliation**	**Edinburgh Napier University, School of Health and Social Care**
**Date**	**16-Feb-2026**
**COI**	**None**

Thank you for the opportunity to review this interesting qualitative paper about physical activity and cardiac rehabilitation after coronary artery dissection. This is an area where care is definitely lacking and so this will be a welcome addition to the literature. I have a few comments that I hope will help you to improve the manuscript before publication:

Introduction

The introduction is well written.

Sampling methods:

This needs more clarity. I understand from what you have written how recruitment via a SCAD support group happened, but please give further detail about how you recruited from hospitals. Was this via a registry or from people who were in hospital at the time? Who approached participants etc.

You state that SCAD diagnoses were verified either through national medical records or patient-provided documentation. Please clarify how SCAD was clarified through national medical records (e.g. did the patient consent to this and were the details from

the SCAD registry or was this from hospital notes because the person was in hospital at the time?)

Page 5 line 17 and data collection sessions

It seems strange to say that interviews were scheduled at mutual convenience when these were focus groups. Does this need rewording as participants would have needed to fit with others?

This is probably a terminology issue, but I find it confusing to use interviews (which indicate to me individual interactions) interchangeably with focus groups. I would suggest revising to focus groups throughout (including the title).

Results

Did you collect data on number of SCADs? This would be interesting to add into the characteristics table if so.

The thematic analysis is very interesting and bring out many issues for this patient group. The results flow well but I think that there is some overlap in themes, particularly around support and advice from healthcare professionals and sometimes about individualised approach. I would encourage you to go back through your themes and make sure that they stand alone. I am not suggesting a reanalysis, just a review to avoid crossover.

Discussion

To a large extent the discussion shows that your findings reflect what is already known in this area. Is it possible to draw out more of what is novel about your study findings?

Reviewer 2

Name O'Regan, Andrew

Affiliation University of Limerick Faculty of Education and Health Sciences, University of Limerick School of Medicine

Date 04-Mar-2026

COI None

I wish to commend the authors on a well written and interesting paper. The topic is likely to be of interest to a clinical as well as academic audience and with that in mind I have the following recommendations:

1. introduction: some statistics regarding the percentage of ACS caused by SCAD, percentage female and average age of onset would be very relevant.

2. Methods: explanation of content analysis, how it differs from thematic analysis and why it is best suited to this research would be helpful.

3. As this research was conducted in Sweden the authors should be explicit in terms of language - the language used for the focus group; if translators were used and any potential 'lost in translation' that translation may cause or alternatively that may occur when interviewing people in a language that is not their first language.

4. Confirm how many people were approached to participate.

5. This is a query and not a criticism. Can two researchers within the same research team provide analytical triangulation. My understanding is that in qualitative research, researchers discuss, debate and often compromise to reach agreement on understanding, i.e. that this is part of the process as opposed to triangulation.

6. Given the broad age range illustrated in Table 1. could the authors insert an age or age band for each participant illustrative quote as this gives contextual information on the quotes.

7. A related point, the presentation of the findings is very much based on consensus of the focus groups but surely there were points on which participants diverged and some had very different experiences to others. Given the age range there must surely have been a range of experiences with physical activity and fitness prior to the SCAD event.

8. In the discussion section a paragraph on implications for practice,policy and research should be included.

9. the phrase 'listen to your body' on lines 9 and 19 of page 17 (discussion section) should be referenced. It does not appear appropriate as it stands in a scientific paper.

10. in the limitations section, the fact that the focus groups were online should be discussed. A recognised strength of focus groups for data collection is the group dynamic and this is likely to be changed in an online setting.

VERSION 1 - AUTHOR RESPONSE

GENERAL COMMENTS	
**Reviewer #1**	
Thank you for the opportunity to review this interesting qualitative paper about physical activity and cardiac rehabilitation after coronary artery dissection. This is an area where care is definitely lacking and so this will be a welcome addition to the literature.	Thank you!
**Reviewer #2**	
I wish to commend the authors on a well written and interesting paper. The topic is	Thank you!

likely to be of interest to a clinical as well as academic audience.	
**INTRODUCTION**	
**Reviewer #1**	
The introduction is well written.	Thank you!
**Reviewer #2**	
Some statistics regarding the percentage of ACS caused by SCAD, percentage female and average age of onset would be very relevant.	Additional epidemiological information has been added to the Introduction to more clearly describe the proportion of ACS attributable to SCAD, as well as the typical sex distribution and age at presentation.
**METHODS**	
**Reviewer #1**	
The sampling methods need more clarity. I understand from what you have written how recruitment via a SCAD support group happened, but please give further detail about how you recruited from hospitals. Was this via a registry or from people who were in hospital at the time? Who approached participants etc.	Additional details have been added to the Methods section to clarify the hospital-based recruitment process. Specifically, we now describe that clinicians at participating hospitals informed patients with a known SCAD diagnosis about the study during routine outpatient visits, and that interested patients were provided with study information and the researchers' contact details. Patients who wished to participate contacted the research team directly.
You state that SCAD diagnoses were verified either through national medical records or patient-provided documentation. Please clarify how SCAD was clarified through national medical records (e.g. did the patient consent to this and were the details from the SCAD registry or was this from hospital notes because the person was in hospital at the time?)	The Methods section has been revised to clarify the verification process. SCAD diagnoses were confirmed through review of medical records following written informed consent from participants. In cases where access to electronic medical records was not possible due to differences in healthcare systems, participants provided copies from their medical journal of relevant medical documentation to confirm the diagnosis.
It seems strange to say that interviews were scheduled at mutual convenience when these were focus groups. Does this need rewording as participants would have needed to fit with others?	The wording has been revised in the Methods section to better reflect the nature of focus group interviews. The sentence now clarifies that interviews were scheduled at times agreed upon by all participants after several alternative dates had been proposed.
This is probably a terminology issue, but I find it confusing to use interviews (which indicate to me individual interactions) interchangeably with focus groups. I would	We agree that consistent terminology improves clarity. The manuscript has been revised to use "focus groups" consistently throughout when referring to the data

suggest revising to focus groups throughout (including the title).	collection method, including in the title, to avoid confusion with individual interviews.
**Reviewer #2**	
Explanation of content analysis, how it differs from thematic analysis and why it is best suited to this research would be helpful.	Thank you for this comment. We agree that several qualitative analytic approaches could have been used. We chose qualitative content analysis since our aim was explore patients' perceptions of physical activity and their experiences of cardiac rehabilitation following the SCAD event, and to stay close to the manifest content of the discussions. Content analysis is commonly used for focus group data and is recommended by Krueger and Casey as an appropriate analytic strategy for systematically categorising participants' expressed views.<br><br>In contrast to more interpretive approaches such as thematic analysis, content analysis allowed us to summarise and structure the participants' perspectives without moving into a higher level of abstraction. We have clarified this rationale in the manuscript and hope this addresses the reviewer's concern.
As this research was conducted in Sweden the authors should be explicit in terms of language - the language used for the focus group; if translators were used and any potential 'lost in translation' that translation may cause or alternatively that may occur when interviewing people in a language that is not their first language.	The focus groups were conducted in Swedish, which is the participants' and researchers' native language. All quotations used in the manuscript were translated verbatim into English by the first author. No external translators were involved.<br><br>We recognise that translation may introduce nuances that are difficult to fully capture across languages. To minimise this risk, the research team discussed the translations to ensure that the English quotations accurately reflected the meaning conveyed in the original Swedish data. Because participants were interviewed in their first language, there was no risk of loss of meaning due to participants expressing themselves in a non-native language.
Confirm how many people were approached to participate.	The Methods section has been clarified to better describe the recruitment process. Participants were recruited consecutively as focus groups were scheduled, and all individuals approached agreed to participate, with one later withdrawing due to illness.

This is a query and not a criticism. Can two researchers within the same research team provide analytical triangulation. My understanding is that in qualitative research, researchers discuss, debate and often compromise to reach agreement on understanding, i.e., that this is part of the process as opposed to triangulation.	We agree that discussion, debate, and negotiated agreement are integral parts of qualitative analysis. In our study, we use the term analytical triangulation to describe the process whereby the initial coding and categorisation developed by the first author were subsequently reviewed, challenged, and refined through systematic comparison with interpretations made by the other researchers.<br>Although the researchers were part of the same research team, they entered the analytic process with different disciplinary backgrounds and analytic sensibilities. The purpose was not to achieve independent coding, but to enhance reflexivity, reduce the influence of a single researcher's perspective, and strengthen the credibility of the analytic claims.<br>This use of triangulation aligns with established descriptions of investigator triangulation, which emphasise the value of multiple analysts contributing to interpretation, even when working collaboratively within the same team (we have now added Carter et al. The use of triangulation in qualitative research. Oncology Nursing Forum, 2014;41:545-547 as a reference).
**RESULTS**	
**Reviewer #1**	
Did you collect data on number of SCADs? This would be interesting to add into the characteristics table if so.	We agree that information on the number of SCAD events could be of interest. However, such data were not collected as part of this study and are therefore not available for inclusion in the characteristics table.
The thematic analysis is very interesting and bring out many issues for this patient group. The results flow well but I think that there is some overlap in themes, particularly around support and advice from healthcare professionals and sometimes about individualised approach. I would encourage you to go back through your themes and make sure that they stand alone. I am not suggesting a reanalysis, just a review to avoid crossover.	We have carefully revisited the thematic structure to assess potential overlap between categories, particularly regarding support, advice from healthcare professionals, and individualised care.<br>We have clarified the boundaries between categories by emphasising that category 3.2 focuses on the content and consistency of information, whereas category 3.3 addresses the organisation and continuity of care. These refinements strengthen the

	distinctiveness of each category without altering the underlying analysis.<br><br>To further enhance clarity, we have made minor revisions to the wording and descriptions of categories where appropriate, in order to better delineate their scope and reduce any potential ambiguity.
**Reviewer #2**	
Given the broad age range illustrated in Table 1, could the authors insert an age or age band for each participant illustrative quote as this gives contextual information on the quotes.	We agree that including participant age or age bands alongside illustrative quotations could provide additional contextual information.<br><br>However, given the rarity of SCAD and the relatively small and geographically defined sample in Sweden, we considered that providing such details in connection with individual quotations could increase the risk of participant identification. To ensure confidentiality and protect participant anonymity, we have therefore chosen not to include age information at the individual quote level.<br><br>We have provided an overview of participant characteristics, including age range, in Table 1 to give appropriate context while maintaining confidentiality.
A related point, the presentation of the findings is very much based on consensus of the focus groups but surely there were points on which participants diverged, and some had very different experiences to others. Given the age range there must surely have been a range of experiences with physical activity and fitness prior to the SCAD event.	We agree that divergent views did occur, but they were typically isolated and did not form patterns across groups, which is why they were not presented as separate categories. Our unit of analysis was the focus group as a whole, and our use of qualitative content analysis aimed to identify the manifest, shared patterns in participants' accounts. While the findings emphasise areas of consensus, this does not mean that all participants had identical experiences.<br><br>We selected quotations that best illustrated the categories and reflected views expressed by several participants in the focus group, rather than long interactional sequences, to maintain clarity and readability.<br><br>We have now clarified in the manuscript that the analysis focused on shared patterns while acknowledging that individual

	variation existed but did not constitute distinct analytic categories.
**DISCUSSION**	
**Reviewer #1**	
To a large extent the discussion shows that your findings reflect what is already known in this area. Is it possible to draw out more of what is novel about your study findings?	We agree that the discussion section needed a clearer articulation of the novel contributions of our study. In the revised manuscript, we have expanded the discussion to more explicitly highlight what is unique in our findings and how they extend existing knowledge.<br><br>Specifically, we have:<br><br>Elaborated on the novel insight that bodily uncertainty after SCAD functions as an ongoing regulatory practice, where patients actively test, interpret, and recalibrate bodily signals in their everyday lives. To our knowledge, this interpretive labour has not been previously described in SCAD research.<br><br>Expanded the discussion of the informational landscape, showing that patients not only receive inconsistent advice but also engage in substantial self-directed “informational work” to construct their own understanding of safe physical activity. This shift of responsibility from healthcare to patient is a new contribution.<br><br>Added a discussion of continuity of care, emphasising the relational dimension of continuity, the need to feel “held” within the healthcare system, which has been underexplored in prior SCAD studies.<br><br>Strengthened the interpretation of peer support, showing that it serves not only as emotional reassurance but as a meaning-making process that helps patients reinterpret bodily sensations and reconstruct a coherent illness narrative.<br><br>Clarified how patients regain control over physical activity through micro-adaptations in daily life, offering new insight into flexible, personalised strategies that fall outside traditional cardiac rehabilitation models.

	These additions are now integrated into the discussion, and together they more clearly articulate the unique contributions of our study to the understanding of SCAD recovery.
**Reviewer #2**	
In the discussion section a paragraph on implications for practice, policy and research should be included.	A paragraph outlining the implications for clinical practice, policy, and future research has now been added to the Discussion section.
The phrase 'listen to your body' on lines 9 and 19 of page 17 (discussion section) should be referenced. It does not appear appropriate as it stands in a scientific paper.	The phrase "listen to your body" reflects advice reported by participants and has now been placed in quotation marks and explicitly contextualised as patient-reported guidance. It is not used as a scientific concept, but as an empirical description of the advice participants received.
In the limitations section, the fact that the focus groups were online should be discussed. A recognised strength of focus groups for data collection is the group dynamic, and this is likely to be changed in an online setting.	Thank you for this important comment. We agree that online focus groups may influence group dynamics compared to in-person settings. In our study, the decision to conduct the focus groups online was deliberate, as SCAD is a rare condition and participants were geographically dispersed across Sweden. The online format enabled participation from multiple sites and made it feasible to gather a sufficient number of participants.<br><br>We acknowledge that online focus groups may alter interactional patterns and group dynamics. In the revised manuscript, we have added a discussion of the potential advantages and limitations of online focus groups, drawing on existing methodological literature.<br><br>We appreciate the suggestion and have now addressed this in the limitations section.
